# Classical dynamin DNM1 and DNM3 genes attain maximum expression in the normal human central nervous system

**DOI:** 10.1186/1756-0500-7-188

**Published:** 2014-03-28

**Authors:** Antoni Romeu, Lluís Arola

**Affiliations:** 1 Nutrigenomics Research Group, Department of Biochemistry and Biotechnology, Rovira i Virgili University, Tarragona, Spain

**Keywords:** Dynamin, Central nervous system, Gene expression, Data mining

## Abstract

Dynamin is a super-family of large GTPase proteins that polymerise during their biological activity. Dynamin polymers form around lipid tubes and contribute to the membrane fission and scission of nascent vesicles from parent membranes. Here we used the NCBI Gene Expression Omnibus (GEO) database and the BioGPS gene expression portal to study differential dynamin gene expression in normal human organs or tissues. From the GDS1096 and GDS596 dataset, we downloaded the relative expression levels of dynamin-related genes (presented as percentages), with respect to all of the other genes on the array (platform Affymetrix GPL96), which includes the best characterised human genes. The expression profiles of dynamin in the central nervous system (CNS) are clearly distinct from the expression profiles in the other organs or tissues studied. We found that the classical dynamin DNM1 and DNM3 genes reach their maximum expression levels (100% of maximal expression) in all normal human CNS tissues studied. This is in contrast to the expression profile in the other normal human organs or tissues studied, in which both dynamin DNM1 and DNM3 genes showed approximately 50% maximal expression. This data mining analysis supports the concept that there is a relationship between the synapse and the molecular function of dynamin, suggesting a new field of work in the study of neurodegenerative diseases.

## Discussion

We have focused our attention on the expression profiling of dynamin genes in several normal human organs or tissues, using the NCBI Gene Expression Omnibus (GEO) database. We have chosen to study dynamin genes because dynamin is a super-family of GTP-binding mechanochemical proteins that are involved in fundamental processes, such as the scission of nascent vesicles from parent membranes and mitochondrial fusion and/or fission [1]. In addition, we believe that the dynamin super-family is a biological system suitable for studying differential gene expression in organs or tissues.

Specifically, we considered the group of genes coding the following protein families within the dynamin super-family [2]: (*i*) the classical dynamins and dynamin-like proteins that tubulate and sever membranes and are involved in clathrin-mediated endocytosis and other vesicular trafficking processes (DNM1, DNM1L, DNM2, DNM3); (*ii*) the guanylate-binding proteins, which are induced by type II interferons and anti-inflammatory cytokines (GBP1 and GBP2); (*iii*) the proteins on the outside of the outer mitochondrial membrane that participate in mitochondrial fusion and contribute to the maintenance and function of the mitochondrial network (MFN1 and MFN2); (*iv*) proteins in the mitochondrial inter membrane space that are involved in mitochondrial fusion, and in which mutations in the gene are associated with optic atrophy type 1 (OPA1); and (*v*) Mx proteins, which are involved in resistance against viral infections (MX1 and MX2). Taken together, these proteins constitute a large set of GTPase proteins that polymerise within each group or family according to the dynamin molecular function [3]. These dynamin polymers (such as rings and helices) are formed around lipid tubes and contribute to membrane fission [4].

**Figure 1:**
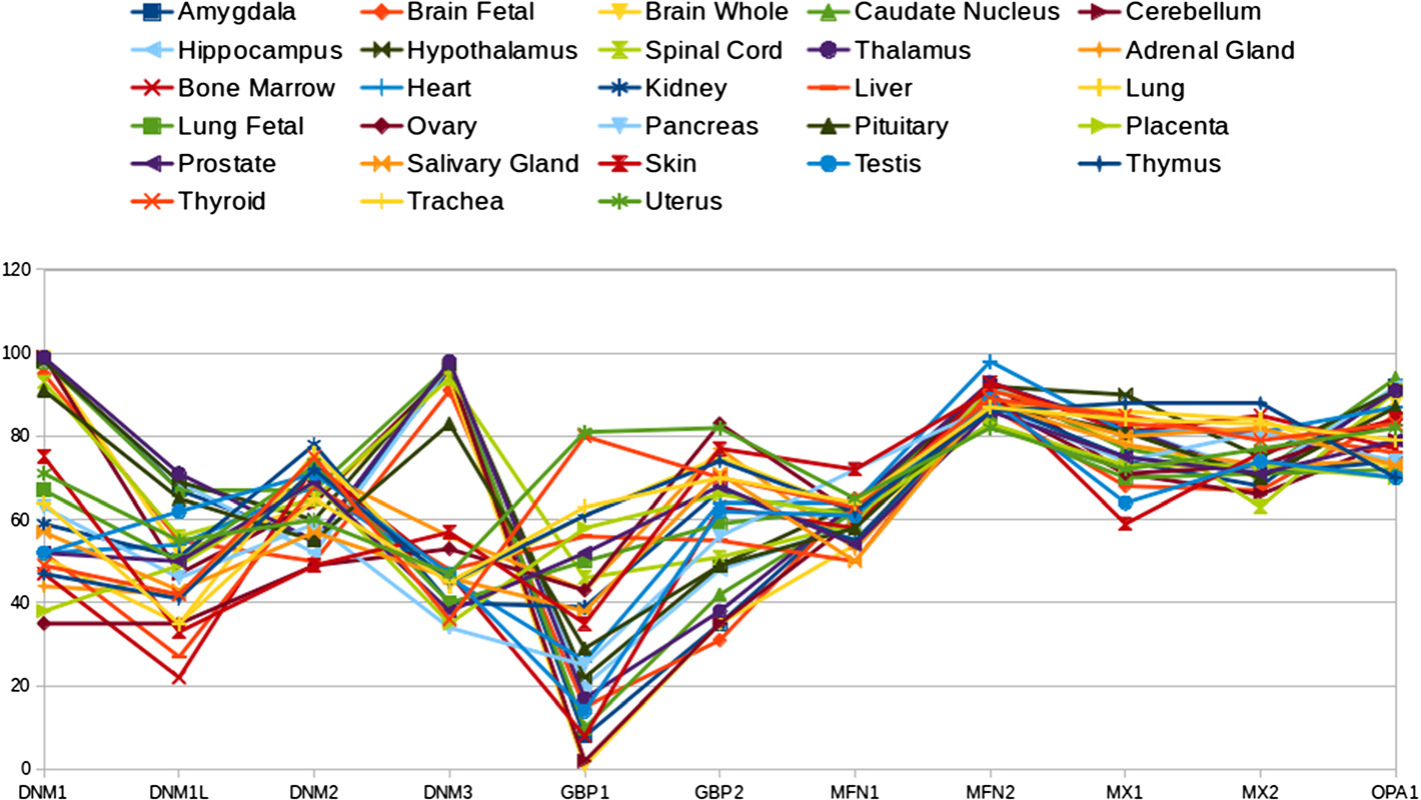
**Data retrieved from the dataset records GDS596 and GDS1096 of NCBI-GEO database.** The figure depicts the dynamin expression profiles of several normal human organs or tissues. The dynamin genes are listed on the x-axis, while, the relative gene expression is shown on the y-axis. The points represent the gene expression level (presented as a percentatge). Each point represents the mean of the gene expression level in normal organ or tissue samples that were common to both of the independent experimental datasets (GDS596 and GDS1096) of the NCBI-GEO database. According to the NCBI-GEO data analysis, all values within an array are rank ordered and then placed into percentile ‘bins’. In other words, all the values of one hybridisation are sorted and then split into 100 groups. Thus, the points give an indication of where the expression of a given gene falls with respect to all genes on that array. In these records, the platform GPL96 (Affymetrix Human Genome U133A Array) were used, that includes over 1,000,000 unique oligonucleotide features covering more than 39,000 transcript variants, which in turn represent more than 33,000 of the best characterised human genes. See Table 1 for a detailed specification of the data.

**Figure 2:**
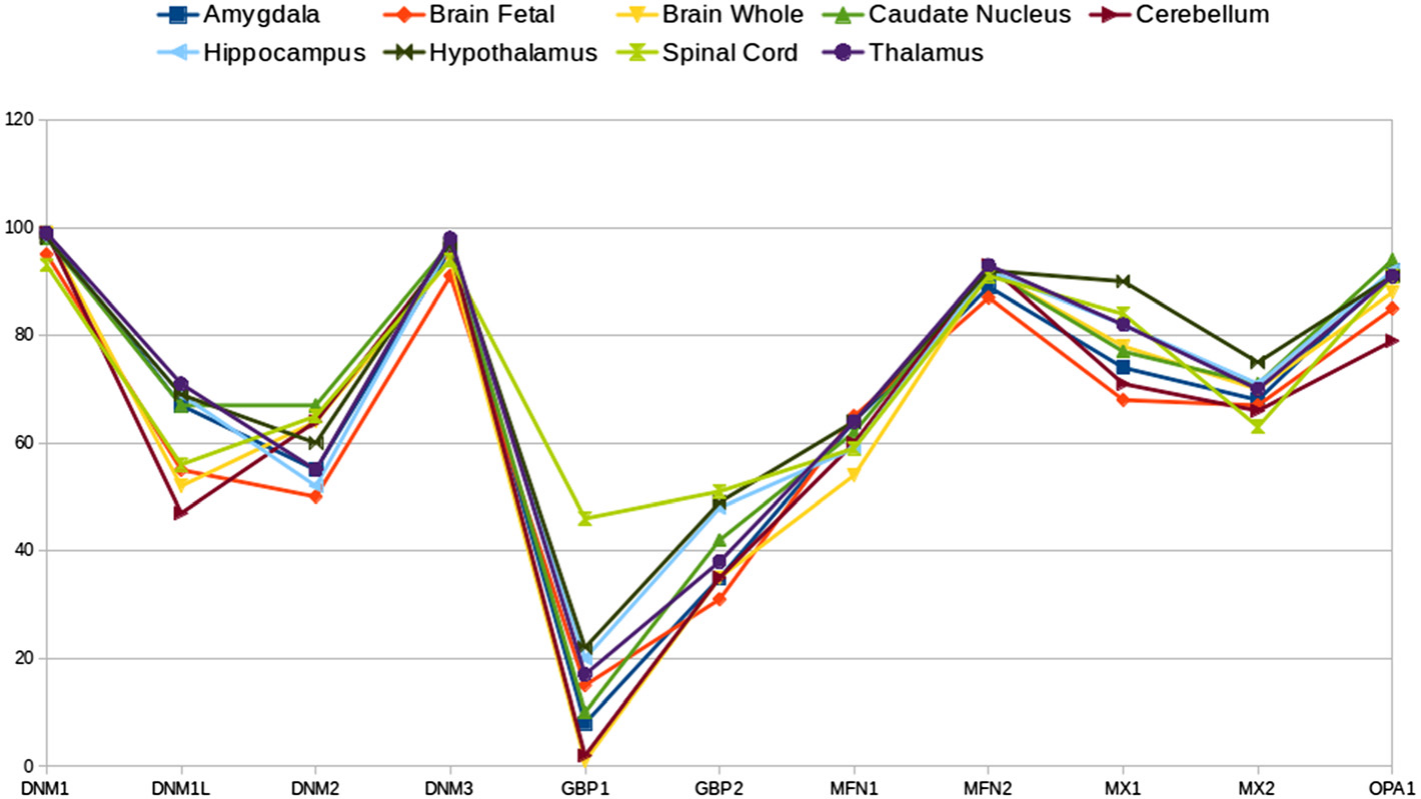
**Dynamin expression profiling in representative components of normal human CNS tissues and organs.** Data retrieved from Figure 1. This figure depicts the expression profiling of the representative components of the CNS. The genes studied are denoted along the x-axis and the gene expression (presented as a percentatge) on the y-axis. The Figure 1 legend explains how to interpret the NCBI-GEO profile charts.

To address this issue, we relied on the independent studies by Su A.I. et al. [5] (GDS596) and Ge X. et al. [6] (GDS1096). We downloaded the relative expression levels of dynamin-related genes (presented as percentages) with respect to all other genes on the array from these NCBI-GEO database records. It is important to note that both NCBI-GEO data sets were created based on the same array platform (Affymetrix GPL96), which include all the best characterised human genes. Figure 1 shows the profiles of dynamin gene expression levels (expressed as percentage and presented as beam shaped lines) in samples from normal human organs or tissues that are common to the NCBI-GEO records of both studies. From a strict visual analysis of these beam lines, the data can be separated into two consistent populations or sub-beam lines of expression profiles, one of which groups together the profiles corresponding to components of the central nervous system (CNS) (Figure 2). This data mining analysis allowed us characterise dymanin gene expression trends in the CNS. We found clear evidence that the classical dynamin DNM1 and DNM3 genes reach their maximum expression level (100% measurement score) in all of the normal human CNS tissues studied. Moreover, this results is in contrast to the expressionprofiles observed in the other normal human organs and tissues studied, in which both the dynamin DNM1 and DNM3 genes showed approximately 50%. A detailed breakdown of the data is presented in Table 1. To improve the rigor of our analysis, we used the BioGPS gene expression portal [7,8] to explore the expression profiles of human dynamin-retaled genes. We found that the classical dynamin DNM1 and DNM3 genes are also maximally expressed in normal human CNS components and at significantly lower level in others organs or tissues [9,10].

**Table 1:** Relative gene expression profiles from normal human organs and tissues (presented as percentatges)

**Organ-tissue**	**DNM1 (215116_s_at)**	**DNM1L (203105_s_at)**	**DNM2 (202253_s_at)**	**DNM3 (209839_at)**	**GBP1 (202269_x_at)**	**GBP2 (202748_at)**	**MFN1 (211801_x_at)**	**MFN2 (201155_s_at)**	**MX1 (202086_at)**	**MX2 (204994_at)**	**OPA1 (212213_x_at)**
Amygdala	**99**	67	55	**96**	8	35	64	89	74	68	92
Brain foetal	**95**	55	50	**91**	15	31	65	87	68	67	85
Brain whole	**99**	52	64	**94**	1	35	54	92	78	70	88
Caudate nucleus	**98**	67	67	**97**	10	42	62	92	77	71	94
Cerebellum	**99**	47	64	**96**	2	35	60	93	71	66	79
Hippocampus	**98**	69	52	**97**	20	48	59	92	82	71	92
Hypothalamus	**98**	69	60	**97**	22	49	64	92	90	75	91
Spinal cord	**93**	56	65	**94**	46	51	59	91	84	63	91
Thalamus	**99**	71	55	**98**	17	38	64	93	82	70	91
Adrenal gland	**44**	42	72	**56**	43	77	53	88	78	73	74
Bone marrow	**47**	22	73	**47**	8	63	58	93	81	85	77
Heart	**52**	54	68	**46**	26	64	64	98	81	81	87
Kidney	**59**	51	78	**40**	39	67	55	88	75	71	74
Liver	**52**	27	68	**48**	56	55	50	91	83	81	76
Lung	**51**	35	76	**44**	61	75	62	88	86	84	72
Lung foetal	**67**	50	73	**40**	50	59	63	83	70	71	72
Ovary	**35**	35	49	**53**	43	83	61	88	71	73	84
Pancreas	**63**	46	59	**34**	25	56	72	87	74	81	74
Pituitary	**91**	65	55	**83**	29	49	58	88	81	70	87
Placenta	**38**	49	69	**35**	58	66	61	83	73	73	70
Prostate	**52**	50	69	**38**	52	68	54	86	75	71	79
Salivary gland	**57**	43	57	**46**	38	71	50	89	80	82	73
Skin	**75**	33	49	**57**	35	77	72	91	59	76	84
Testis	**52**	62	71	**47**	14	62	61	89	64	74	70
Thymus	**47**	41	72	**45**	61	74	62	86	88	88	70
Thyroid	**49**	42	75	**36**	80	70	63	89	85	79	83
Trachea	**64**	35	65	**45**	63	70	64	87	84	83	79
Uterus	**71**	55	60	**47**	81	82	65	82	72	77	82

Data are presented as the mean values of the dynamin gene expression percentatges calculated from the individual values of the samples common to both NCBI-GEO records (GDS596 and GDS1096). The GPL96 Affymetrix probe set ID is displayed in parentheses below the gene symbol. Where there is more than one entry for the same gene, we selected the entry for which the Affymetrix probe set ID refers to the mRNA or coding DNA sequence (cds). The CNS tissues and organs are shown in the top block of the table. DNM1 and DNM3 gene expression values are shown in bold. See the legend of Figure 1.

The CNS consists of the brain and spinal cord, where synapses are the dynamic structures through which all nervous system signals traverse. Dynamin polymerisation in membrane fission is thought to play a significant role in the synapse (Gene Ontology GO:0003373) which is suggestive of the concept of a relationship between synapses and dynamins [11]. Several animal model studies have shown that dynamin genes are highly expressed in neurons [12-15]. In conclusion, based on their high levels of expression in human CNS, DNM1 and DNM3 can be considered as CNS-specific dynamin genes, at least in the following human CNS components: amygdala, foetal brain, whole brain, caudate nucleus, cerebellum, cerebellum peduncles, cingulate cortex, globus pallidus, hippocampus, hypothalamus, medulla oblongata, occipital lobe, parietal lobe, pineal day, pineal night, pons, prefrontal cortex, spinal cord, subthalamic nucleus, temporal lobe, and thalamus. We suggest that the role of dynamins in the CNS could be a potentially interesting area for further biochemical research in neurodegenerative diseases.

## Competing interests

The authors declare that they have no competing interests.

## Authors’ contributions

AR carried out the downloading of gene expression data from the NCBI-GEO database, helped design the study, and contributed to writing the manuscript. LA wrote the manuscript and helped design the study. Both authors read and approved the final manuscript.
